# Identifying the Mechanism of Interaction Between Soil Moisture State and Summertime MCS Initiations in Weakly Forced Synoptic Environments Using Convective‐Permitting Simulations

**DOI:** 10.1029/2024JD040855

**Published:** 2024-12-01

**Authors:** Rachel Gaal, James L. Kinter, Paul A. Dirmeyer, Bohar Singh

**Affiliations:** ^1^ Department of Atmospheric Science Rosenstiel School of Marine and Atmospheric Science University of Miami Miami FL USA; ^2^ Center for Ocean–Land–Atmosphere Studies George Mason University Fairfax VA USA; ^3^ Climate Prediction Center National Oceanic and Atmospheric Administration College Park MD USA

**Keywords:** land‐atmosphere interactions, mesoscale convective systems, convective‐permitting simulations, soil moisture‐precipitation coupling, US Great Plains

## Abstract

This work aims to identify a mechanism of interaction between soil moisture (SM) state and the incidence of weakly forced synoptic scale MCS events during boreal summer by performing a sensitivity study using the Weather Research and Forecasting (WRF) model over the US Great Plains. A uniformly dry SM patch at a 5° × 5° scale is centered at the point of a documented MCS initiation to observe spatiotemporal changes of the simulated MCS events, totaling 97 cases between 2004 and 2017. A storm‐centered composite analysis of SM at the location of simulated MCS events depicted SM heterogeneity [O(100) km] structured as significantly drier soils to the southwest (SW) transitioning to wetter soils northeast (NE) of the mean simulated initiation. Further analyses showed that this SM configuration influenced near‐surface fluxes, which created a gradient of 2m‐temperature and 2m‐humidity, also aligned SW‐to‐NE, which affected the growth of the planetary boundary layer to trigger MCS initiations earlier in time (∼1–2 hr on average) compared to Control simulations. The implementation of the dry SM perturbation introduced drier‐to‐wetter SM gradients along the edges of the perturbed area, and MCS initiations were subsequently preferred on the drier side of those transition zones, with the most common orientation of simulated MCS events embedded within southwesterly flow. These results emphasize the importance of the low‐level wind field alignment to organized SM gradients, which suggests that SM heterogeneity can drive MCS initiation related to near‐surface atmospheric variable fluctuations as the main mechanism of interaction in weakly forced synoptic environments.

## Key Points

- Perturbating soil moisture initial conditions in a convective‐permitting model influenced the timing and location of simulated MCSs
- MCS events occurred earlier (∼1–2 hr on average) than Control simulations
- On average, initiations were detected on the dry side of soil moisture gradients with low‐level winds aligned parallel to gradient direction

## 1 Introduction

The mesoscale convective system (MCS) is the largest form of organized deep convection on Earth, described as an aggregate of cumulonimbus clouds often characterized by its large upper cirriform cloud structure that reaches the meso‐α length scale (200–1,000 km; Houze, 2004, 2018). The combination of larger, longer‐lasting stratiform precipitation and concentrated, heavier periods of convective rainfall relates the MCS with localized flash flooding (Schumacher & Rassmussen, 2020), accompanied by other severe weather hazards such as straight‐line winds and hail (Houze, 2004; Houze et al., 1990). Because of the length scales at which the MCS occurs, understanding the spatiotemporal characteristics of the synoptic‐scale atmosphere (referring to length scales spanning 1,000 km) becomes increasingly important and has become the focus of recent research that aims to increase the overall predictability of these events.

Although MCSs are observed in many areas around the world, the United States (US) has a unique topography that creates a region wherein MCSs most commonly form. The direction of the large‐scale atmospheric flow allows for mid‐tropospheric perturbations to propagate over the Rocky Mountains, initiating convection on the leeward side in late‐day hours (Wang et al., 2011), while abundant moisture is funneled into this same region by advection by the Great Plains low‐level jet (GPLLJ) (Higgins et al., 1997). Feng et al. (2019) characterized the salient atmospheric features for MCS events occurring between 2004 and 2016 east of the Rocky Mountains, which were categorized by the seasonality, synoptic‐scale forcing strength, and region. For summer events over the US Great Plains, it was found that MCS events frequently occur in weakly forced synoptic environments, featuring a high‐pressure ridge just east of the initiation point. Sufficient (although smaller anomalies compared to other seasons) low‐level moisture transport by the GPLLJ was present, associated with warmer temperatures in the summer, which is consistent with other findings in the same geographical region (Song et al., 2019). Although the mean thermodynamic environment in this case is considered favorable, the lack of positive anomalous dynamic atmospheric properties has led both Feng et al. (2019) and Song et al. (2019) to further suggest that smaller scale perturbations are responsible for the triggering of MCS in weakly forced synoptic environments, and that summer MCSs in the Great Plains region might be inherently less predictable due to the frequency in which these events occur in weakly forced synoptic environments.

A form of smaller scale perturbations linked to deep convection onset, specifically in the Great Plains region, is the interaction of soil moisture (SM) anomalies with the atmosphere, where SM content directly influences the partitioning of latent and sensible heat fluxes at the surface, modifying near‐surface temperature and moisture and subsequently thermodynamic properties of the atmosphere (e.g., Ford et al., 2014; Seneviratne et al., 2010). These relationships, often defined in the framework of land‐atmosphere (L‐A) interactions (e.g., Koster et al., 2004; Santanello et al., 2011), are best described by varying positive and negative feedback loops wherein SM state modulates the anomalous occurrence of clouds and subsequent precipitation (P), and hence the framework known as SM‐P coupling. Geographic zones of strong SM‐P coupling are defined as “hot spots” (Koster et al., 2004), which are characterized by transitional zones between arid and humid climates. These transitional zones are unique areas where evaporation is high but still sensitive to SM, and there is enough boundary layer moisture to trigger convection (Seneviratne et al., 2010; Sud et al., 1993). The US Great Plains has repeatedly been shown to be considered a L‐A hot spot (e.g., Ferguson et al., 2012; Santanello et al., 2013), situated in the transition zone from western (arid) to eastern (humid) regions.

Therefore, the probability of detecting a relationship between SM state and the onset of deep convection in this geographic region is high; however, SM‐P coupling in this region is complicated in that conflicting results exist: some studies suggest a wet‐soil feedback, meaning increased precipitation over wetter than average soils (e.g., Gerken et al., 2018; Guillod et al., 2015), and both wet‐ and dry‐soil feedbacks have been shown to exist depending on the Great Plains sub‐region (Alfieri et al., 2008; Ford, Rapp, & Quiring, 2015; Ford, Rapp, Quiring, & Blake, 2015; Ford et al., 2023). This lack of consensus causes confusion insofar as predictability and model fidelity are dependent on the sign and strength of the SM‐P relationship (Ford, Rapp, & Quiring, 2015; Ford, Rapp, Quiring, & Blake, 2015; Ford et al., 2014, 2018; Simon et al., 2021; Yuan et al., 2020). It is also illustrated in these aforementioned studies that synoptic‐scale forcing affects the strength and sign of the SM‐P relationship. For example, Ford, Rapp, and Quiring (2015) and Ford, Rapp, Quiring, and Blake (2015) took into consideration the synoptic‐scale atmospheric conditions when assessing the presence and sign of SM‐P coupling in Oklahoma and found that a dry‐soil feedback dominates when ridging at the 500 hPa level occurs. Welty and Zeng (2018) found that partitioning deep convective cases occurring in the southern Great Plains by water vapor convergence values (used to describe the strength of synoptic‐scale forcings) also showed a dry‐soil feedback when water vapor convergence values were low, but a wet‐soil feedback in opposing dynamic conditions and decreasing feedback strength overall with no synoptic‐scale‐forcing partitioning.

Organized convective events such as the MCS are associated with stronger synoptic‐scale forcings (i.e., passing frontal boundaries) that dominate as the lifting mechanism over smaller scale perturbations defined in the L‐A framework. Therefore, there is a smaller subset of literature that looks directly at the relationship between MCS initiation and SM state, but there are diverse findings in that the strength and sign of the relationship is dependent on geographic region, and the proximity of differing SM values to one another in space (SM heterogeneity).

In the African Sahel, Taylor et al. (2011; hereafter T2011) showed that SM heterogeneity (at length scales between 10 and 40 km) influenced the location and timing of afternoon MCS rainfall by doubling the probability of convection if the SM values were aligned with the surface winds such that dry anomalies were positioned upstream and wet anomalies were positioned downstream. Klein and Taylor (2020) focused on the effects of SM on MCS development and found an enhancement of MCS (through intensification of convergence, wind shear, instability, and moisture) downstream of anomalously dry patches of SM [O(200 km), which refers to dry patch length scale]. Gaal and Kinter (2021; hereafter GK2021) also found that SM heterogeneity played a role in the placement of MCS initiations in the US Great Plains. GK2021 found the antecedent SM and 2m‐temperature (T2m) gradients were aligned with surface winds, such that anomalously dry SM patches (O[100 km]) were located upstream and anomalously wet SM patches were located downstream.

The SM gradients described in T2011 and GK2021 are the basis of the mechanism of interaction between SM state and the near‐surface atmosphere, where the associated T2m gradients from the SM configuration generate a pressure gradient over the area of heterogeneity, inducing a near‐surface circulation at the meso‐β scale. Like the sea‐breeze, this concept was described in Segal and Arritt (1992) and was further tied to the incidence of unorganized convection (e.g., Froidevaux et al., 2014; Garcia‐Carreras et al., 2011). However, T2011 and GK2021 assessed only observed SM conditions antecedent to MCS initiations, so they could not definitively identify the mechanism of interaction.

Baur et al. (2018; hereafter BKC2018) performed a sensitivity study to address this mechanism of interaction using convection‐permitting simulations of various deep convective events in central Europe, and found that when implementing a dry‐to‐wet checkerboard pattern at varying scales (between 30 and 140 km in length scale), thermally induced circulations driven by sensible heat flux gradients at the meso‐β scale were present (the circulations were most dominant at the 40–80 km pattern scale), with updrafts present over drier SM values (downstream of the wet SM patches). Furthermore, they found that this relationship was only prevalent when considering cases that occurred in weakly forced synoptic environments, further underpinning the importance of partitioning samples based on this criterion. BKC2018, however, is not necessarily focused on MCSs.

Within the existing literature, the main information gap lies within the choice of analyzing stochastic convection versus organized deep convection (such as the MCS), and whether or not strong or weak synoptic‐scale forcings are considered when sampling cases. Specifically attempting to connect SM‐P coupling to the initiation of MCS events in the US Great Plains region, it is implied that SM heterogeneity is prevalent on average in the vicinity [O(100 km)] of select MCS events sampled from observations (i.e., GK2021), but there has yet to be definitive evidence of noncausal link between the underlying SM pattern of weakly forced MCS initiations.

Therefore, the main goal of this work is to perform a sensitivity study with convective‐permitting simulations over the US Great Plains to understand the mechanism of interaction between SM state and the incidence of weakly forced synoptic scale MCS events during boreal summer. The methodology of this study is motivated by the findings of BKC2018 and GK2021, where dry SM patches at the spatial scale of influence found in GK2021 are centered at the point of select MCS initiations, creating a SM gradient at the boundaries of the SM perturbation. The aim of this experimental setup is to observe the effect of this SM perturbation on the location and timing of MCS events comparatively to Control simulations, and whether or not a mechanism can be identified that supports the hypothesis that the alignment of the background wind to a SM heterogeneity pattern (such that dry patches are upstream and wet patches are downstream) induces convergence and upward motion through meso‐β circulations to support the initiation of weakly forced MCS, further supporting the hypothetical mechanism outlined in studies such as T2011 and GK2021. Section 2 focuses on the numerical model of choice and its configuration, Section 3 discusses the experimental methodology, Section 4 highlights data usage, Section 5 explains the main findings of the experiments, and Section 6 summarizes the overall study, along with uncertainties and implications the results have on future work.

## 2 Model Choice and Configuration

### 2.1 The WRF Model

Performing a sensitivity experiment in this context requires a numerical weather model that can best represent both the behavior and characteristics of MCS, and salient atmospheric conditions. Single column models are typically not large enough to simulate MCS events, but global atmospheric models are run at coarser resolution and therefore use convective parameterization (CP), which typically does not resolve meso‐scale circulations that are important components of the MCS. Therefore, a limited area model that can explicitly resolve convection is necessary.

**Figure 1:**
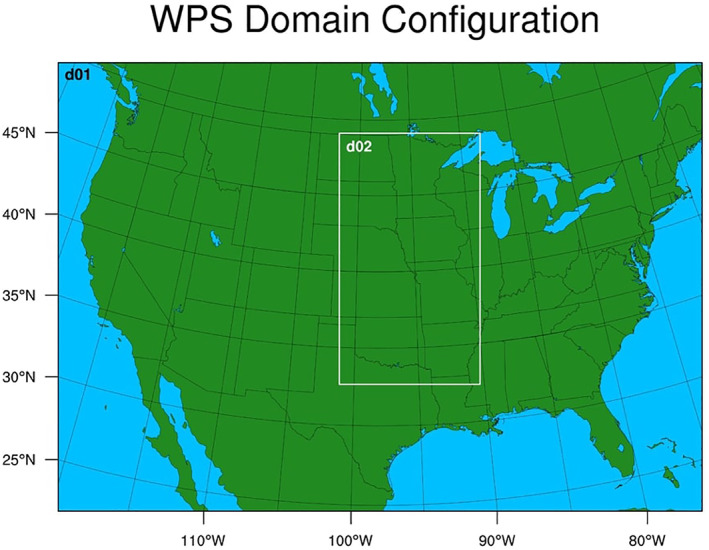
Geographical representation of parent (d01) domain (20 km resolution) and nested (d02) domain (4 km resolution), which includes a portion of the central US (approximately 102°W–90°W, 33°N–49°N).

This study, aimed at simulating the behavior of MCS events in the presence of idealized and observed SM distributions, employs the Weather Research and Forecasting (WRF) model version 4.3.1 (Skamarock et al., 2021). WRF has been used to simulate MCS in the US Great Plains region (e.g., Feng et al., 2018) and is a highly customizable model in terms of the parameterization combinations available for use. The particular setup for this experiment uses WRF to perform two‐way, two‐input nested runs, with one parent domain (d01; 20 km resolution) and one nested domain (d02; 4 km resolution), depicted in Figure 1. In this configuration, both the parent and nested domain communicate simultaneously with one another, providing updated fluxes and state variables after each time step. The choice of 4 km grid spacing is based on both recommendations from the parent‐grid ratio in WRF documentation (UCAR, 2023) and for use in shorter time frame forecasts (Schwartz et al., 2009).

### 2.2 Model Parameterization Choices

Both d01 and d02 are identically configured with the parameterizations listed in Table 1 (excluding the CP scheme, which is turned off in d02). The primary reasoning for this combination of choices is to be as consistent as possible with a well‐tested suite of physics options available to WRF users for this geographic region and purpose of simulation. The “CONUS” physics suite, cataloged in WRF documentation (UCAR, 2023), is intended to be used within a domain configuration that explicitly resolves convection in the nested domain and a parent domain that uses a cumulus physics scheme and has been used in both NCAR real‐time forecasting experiments and the NCAR ensemble prediction system, while also being evaluated for its performance of predicting MCS in the Central US (Clark et al., 2012; Powers et al., 2017; Schwartz, Romine, Sobash, et al., 2015; Schwartz, Romine, Weisman, et al., 2015).

**Table 1:** Physics Parameterizations Listed for Parent (d01) and Nested (d02) Domains Used in Numerical Experiments

Physics scheme	d01—20 km	d02—4 km	Citation
Microphysics	Thompson et al.	Thompson et al.	Thompson et al. (2008)
Cumulus	Tiedtke	–	Tiedtke (1989)
LW/SW Radiation	RRTMG	RRTMG	Iacono et al. (2008)
Surface Layer	Eta similarity	Eta similarity	Janjic (1996)
Land Surface	Noah Land Surface Model	Noah Land Surface Model	Chen et al. (1997) and Ek et al. (2003)
Planetary Boundary Layer	Mellor‐Yamada‐Janjic	Mellor‐Yamada‐Janjic	Janjic (1994)

## 3 Experimental Methodology

The focus of these numerical experiments is to introduce idealized SM in the vicinity of various MCS events to test the sensitivity of MCS initiations in both space and time, as well as to observe the atmospheric response to the SM perturbation by evaluating significant differences between the unaltered (Control) run and each experimental run. The experimental configuration consists of multiple components, including case selection of MCS events, the initialization data sources and verification of WRF, the tracking of MCS initiations in WRF simulations, and the application of the SM experiment.

### 3.1 Soil Moisture Perturbation Experiment

Inspired by the experimental setup of BKC2018, combined with the main findings from GK2021 and T2011, one generalized experimental configuration was created to use in each observed MCS case in the selected sample (*n* = 97) such that the initiation location is used as a center point for a 5° × 5° domain wherein SM is significantly reduced (referred to hereinafter as PB‐1). Although the most influential scale from BKC2018 was reported to be between 40 and 80 km (and up to 40 km in T2011), the scale used here reflects the main findings of GK2021 mainly due to the similarities of area study (i.e., the Great Plains region).

**Figure 2:**
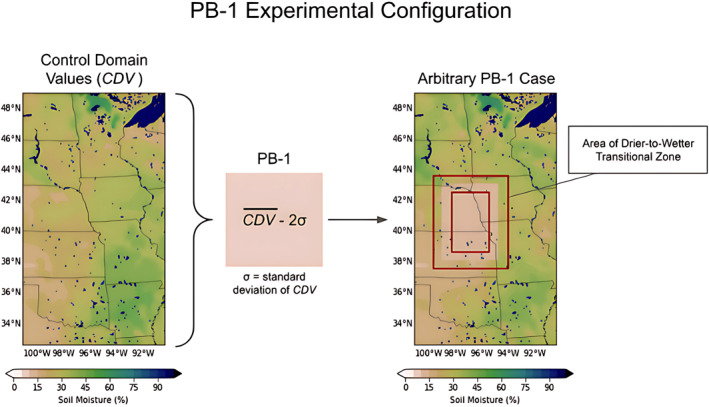
Schematic of PB‐1 experiment with relation to the Control experiment at the initialization time, where $\bar{\text{CDV}}$ stands for Control domain values and the mean control domain value is denoted by ($\bar{\text{CDV}}$). The location for the PB‐1 experiment is for an arbitrary MCS case. Water bodies are denoted by blue shades on the color bar (SM = 100%).

Rather than introducing a checkerboard pattern of varying drier and wetter SM spanning d02 (as in BKC2018), one single patch was perturbed while keeping the rest of d02 at observed values, which is intended to reduce the SM uniformly to a single value near wilting point everywhere in the experiment area. The process for setting up the PB‐1 experiment is schematically shown in Figure 2, where the plotted WRF variable is volumetric SM (SMOIS; m^3^/m^3^) expressed as percentage. The mean control domain value ($\bar{\text{CDV}}$), defined as the mean SM value in d02, and the standard deviation of d02, are calculated to determine the value of the PB‐1 experiment ($\bar{\text{CDV}}$—2σ), which is then implemented into the observed SM at the specified position for the respective case. Different MCS cases have different convective initiation locations, so the SM perturbation, which is applied where the MCS initiation occurred in the observations, occurs at different locations in d02.

The choice to use a near‐wilting point value is to accommodate the fact that the wilting point will vary depending on the dominant soil and vegetation type in the region of each PB‐1 experiment; using a value based on the d02 parameters (mean and standard deviation of d02 SM) takes into consideration the entire domain and its values across all land surface types regardless of event, and therefore can vary on the spread of values for each specific case, providing a more standardized approach of determining PB‐1 SM for the Great Plains region.

An example of what the PB‐1 experiment looks like imposed into arbitrary initial conditions is shown in the right‐hand panel of Figure 2, emphasizing both that the PB‐1 experiment is a large‐scale dry SM area in d02, and also that this expansive dry SM creates drier‐to‐wetter SM gradients along the edges of the implemented experiment. This is to test two things: first, to see if the drying of soil in the vicinity of the observed MCS event has any effect on the spatiotemporal characteristics of the MCS, and second, to test the influence of a drastic dry‐to‐wet SM heterogeneity pattern on MCS initiation location (as hypothesized in GK2021). To illustrate how much the preferred mean SM conditions for MCS initiations vary across the selected cases, Figure S1 in Supporting Information S1 shows the mean SM distributions for each case group, which depicts the large variability of SM that is introduced into the model during the PB‐1 experiment. It should be noted that the SM in the PB‐1 experiment is only applied in the initial conditions and allowed to evolve during the duration of each run. This perturbation also spans the entirety of the soil column (a default of *n* = 4 layers in the Noah LSM).

### 3.2 Initialization Setup

Land boundary and atmospheric data conditions used for initialization were taken from the ECMWF ERA5 Reanalysis (Hersbach et al., 2020). Having a 31 km global spatial resolution and hourly output, this reanalysis product was chosen for the similarities of its spatial resolution to that of d01 and the desired temporal resolution of each simulation. In reference to this particular experiment, the ERA5 is also known to have a more balanced representation of evaporation, precipitation, and subsequently SM, making it relevant for use in this study (Horton, 2022).

The experimental setup choices made in this study were made to best address several factors, including proper spin‐up time, run duration and initialization time, and balancing the time of interaction between perturbed SM (PB‐1) and the early morning atmosphere during the simulation. With reference to spin‐up time, this has been heavily debated and is dependent on a number of factors (e.g., parameterization choices, resolution of boundary and initial conditions, and the role of the synoptic‐scale forcing on simulated event of interest (Liu et al., 2023)), but for limited area models it has been cited to range between 6 hr (Cha & Wang, 2013) and 12 hr (Jankov et al., 2007) in order for the boundary conditions to establish physical equilibrium. An initialization time of 1200 UTC (6a.m. LST) on the day of the observed initiation was chosen for all cases and each simulation was integrated through 24 hr with hourly temporal output available to best estimate the MCS initiation. Although each case has a different observed initiation time, a uniform model initial condition time was used to allow for the implemented SM conditions to interact with the atmosphere in the early morning hours and to allow for the least amount of time for each SM experiment to evolve from its initial values at *T* = 0. It is also ideal to reach the minimum spin up time for each weakly forced event, which we consider to be 6 hr, as most of the cases in the *n* = 97 sample start after 1800 UTC.

### 3.3 Case Selection

The observed MCS cases in this study are chosen from the same MCS database used in GK2021. Originally published by Feng et al. (2019), this case catalog (MCS2019) includes tracked MCS events in the contiguous US east of the Rocky Mountains during 2004–2017. The criteria for case selection in GK2021 is applied to MCS2019 (see Figure 1 in GK2021), which includes choosing only boreal summer events that initiate during local daytime hours (1200–2300 UTC) and are below 1,200 m surface elevation in the US Great Plains. The nested boundary for the Great Plains region in this experimental setup is smaller than the area of interest in GK2021, therefore only initiations occurring well within the nested domain (distance ≥ 2.5° from d02 boundary) are considered. A Convective Triggering Potential—Low‐level Humidity Index (CTP‐HI_low; Findell & Eltahir, 2003a,b) analysis is then performed, which evaluates early morning atmospheric humidity and temperature profiles to identify whether or not the land surface is most likely to influence afternoon convection. Identical to GK2021, only MCS initiations that are not categorized as “atmospherically controlled” are used in the subsequent analysis, which uses major/minor axis length and eccentricity values to classify each case “line/squall” or “non‐line” storms. Because “line/squall” storms are likely associated with strong atmospheric forcing (e.g., fronts), we only include “non‐line” classified cases. This totals 97 samples, with *n* = 30, 27, and 40 cases for June, July, and August, respectively.

Nonevent days (hereafter NoMCS) are also considered, primarily used to verify that WRF is not over‐producing events. Using the NOAA National Centers for Environmental Information (NCEI) Daily Summaries GIS Map Viewer (Menne et al., 2012), 24‐hr periods were evaluated through visual examination to assess daily precipitation values within the boundaries of d02 for all boreal summer days between 2004 and 2017. In order to be considered, the inner domain (d02) did not receive substantial rainfall (<2.54 mm; the smallest threshold in the NCEI Daily Summaries GIS Map Viewer) within the period of sampling (24‐hr). To further verify no large‐scale precipitation occurred during the 24‐hr period of interest, the NOAA Storm Prediction Center (SPC) storm report archive (https://www.spc.noaa.gov/archive/) was used to verify that there were no watches or warnings during these times. Within this evaluated time period, a total of *n* = 14 cases qualified as valid nonevent days.

### 3.4 Identification and Tracking of MCS

To detect MCS events and track their duration and position in all simulations, a modified version of the multiple object tracking algorithm (hereafter MOTA) used in Singh and Kinter (2020) is implemented. Originally used for detecting tropical intraseasonal oscillations from outgoing longwave radiation anomalies, MOTA is based on the prediction‐correction method using a Kalman filter and was augmented in this study to use a size threshold (>6 × 10^4^ km^2^) and brightness temperature threshold (*T*_*b*_ < 241K) as a proxy for MCS initiation, which follows from the classification in Feng et al. (2019; “the first hour when a CCS [cold cloud shield] is detected”). Each track is only considered an MCS event if the CCS persists for at least 6 hr and is tracked until the size threshold is no longer met.

Training MOTA consists of using a random sample of 20 cases from MCS2019, with brightness temperature represented through the National Centers for Environmental Prediction/Climate Prediction Center (NCEP/CPC) Half Hourly 4 km Global Merged IR Data set (Janowiak et al., 2017). This data set was chosen for the similarities of its spatial and temporal resolution to the WRF output data. Successful tracks equated to a detected event happening within 2.5° (and no more than 3 hr time difference) from the MCS2019 initiation point. MOTA was also applied to the NoMCS group to verify that WRF is not unrealistically producing MCS events during atmospheric conditions originally not conducive to large‐scale convection.

### 3.5 Verification of WRF

With these configuration choices, the overall performance of WRF is assessed to verify the validity of the results herein. MOTA was used on the Control runs for all cases (*n* = 97) to track the location in space and time of the simulated versus observed MCS initiations. Table 2 shows this data based on the setup of a contingency table. The number of misses includes two categories: a true miss (i.e., no detected event) and an inaccurate miss (i.e., >2.5° or >3 hr from the observed initiation). A correct negative is calculated by running the simulations with the same configurations for the NoMCS cases and implementing MOTA to assess whether a detectable MCS occurred during the run. Table 2 shows that the model does not produce any events during these days (correct negative = 14).

**Table 2:** WRF Forecasts Compared to MCS2019 Observations, Where the Asterisks (*) Denotes the Summation of True Misses and Inaccurate Misses

		Validation (observed event from MCS2019)	
		Yes	No
WRF Control Forecast	Yes	Hits (58)	False Alarms (0)
	No	Misses (39)* *(No Event = 12; Inaccurate Event = 27)	Correct Negative (14)

## 4 Data Analysis Methods and Framework

After the successful verification of WRF, each MCS case was initialized and performed, then MOTA was used to track the MCS initiations in each PB‐1 experiment. These initiations are then cataloged and compared to the Control case set. With respect to Table 2, 9 case dates out of the 12 true misses documented in the Control runs are also missed in PB‐1, with 3 unique misses in PB‐1 that are not seen in Control. All other cases were equally detected in between case groups. To further investigate the atmospheric, near‐surface, and land surface variable changes, each experiment is examined through two different methods of data analyses (Section 4.1) and the variables are then evaluated for significance (Section 4.2).

### 4.1 Storm‐Centered Composite Analysis and Superposed Epoch Analysis

For this study, the use of bounding boxes centered at the mean latitude and longitude of each recorded MCS initiation in WRF, aligned with constant latitude and longitude, referred to here as storm‐centered composites. Each composite was formed at a 5° × 5° scale (hereafter MCSarea), which was chosen to align with the same scale of the implemented PB‐1 experiment and to evaluate the same area that was found to have the most significant SM heterogeneity pattern in GK2021.

Depending on the variable of interest, the storm‐centered composites are assessed at different times or over periods of time, including morning antecedent hours (1300–1600 UTC), 1hr prior to initiation (Initiation—1 hr), and at the time of the MCS initiation (Initiation). Each evaluated time frame is chosen for specific purposes: morning antecedent hours are used for surface and near‐surface variables mainly due to the influence of the land surface at these hours being greatest on the development of convection (Findell & Eltahir, 2003a). This timeframe is also useful because the possibility of rainfall generated by MCS activity is not likely to influence the state of the land surface antecedent to initiation. Looking just before initiation is mainly used for atmospheric wind variables to assess the structure before meso‐scale circulations associated with MCS development are formed. The SM variable at initiation time is also used to evaluate what the atmosphere is “feeling” in terms of the land boundary conditions at the exact time of initiation.

Another method of viewing near‐surface and atmospheric variables is through a superposed epoch analysis (SEA; Adams et al., 2003), where the evolution of variables with respect to initialization time (1200 UTC) is assessed through time, taking the zonal mean of a storm‐centered composite to illuminate the latitudinal and temporal dependence of the variables. Combined with differencing, the use of SEA can produce plots that derive departures from Control. This method is used in conjunction with atmospheric profile averaging to ultimately produce time‐height plots for those variables that vary with height.

### 4.2 Evaluation of Significance

Comparing the variables from the PB‐1 experiment case to Control also includes an evaluation of whether the variables of interest are significantly different from one another. A two‐tailed Welch's *t*‐test is performed at the 95% level for storm‐centered composites and SEA. The *t*‐test assumes unequal variances and that the null hypothesis states that the two sample means are equivalent. The variables being evaluated, however, are typically non‐Gaussian in nature (i.e., spatially and temporally correlated), and Welch's *T*‐test assumes that variables are sampled from a Gaussian distribution, possibly impacting the confidence estimates. Despite this, the main objective of this study is to highlight the largest existing differences between the PB‐1 experiment and Control, and a *t*‐test is the most straightforward way to highlight this in the analyses herein.

## 5 Results

Although it is expected that the initial perturbation will alter different near‐surface and atmospheric components, the overall aim of this study is to answer the following questions:

1. What is the average SM configuration surrounding the recorded points of MCS initiations in Control and the PB‐1 experiment group?
2. What are the thermodynamic/dynamic pathways between this observed SM state and MCS initiations, and how does that translate to the spatiotemporal differences between MCS initiations in Control versus PB‐1 experiment groups?
3. Can a mechanism of interaction be established like that outlined in GK2021 and T2011?

The results are split into 3 Sections 5.1, 5.2, 5.3 that explain all relevant analyses to better address these questions in detail.

### 5.1 Soil Moisture State Comparison

**Figure 3:**
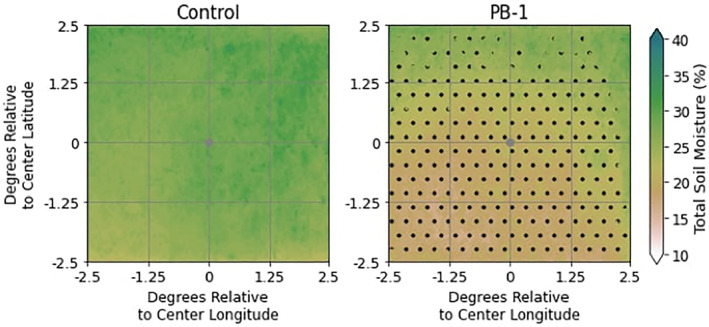
Total SM (between 10% and 40%) storm‐centered composites (centered in space and time at each simulated MCS initiation point) evaluated at initiation time, where significant SM compared to the Control sample is stippled at the 95% confidence level.

To recall the methodology, the EXParea location is determined by the observed (MCS2019) initiation location. It is worth noting, however, that the precise times and locations of convective initiation for the PB‐1 experiment could be different from both observations and Control, creating three sets of coordinates. This establishes the notion that the MCSarea (defined earlier as the 5° × 5° area of analysis surrounding the recorded initiation point) could encompass all, none, or some of the SM perturbation implemented in WRF. To investigate where MCS initiations occurred with respect to underlying SM conditions, Figure 3 shows the average composites of the total SM (%) in the MCSarea for both Control and PB‐1, where each panel shows the MCSarea centered on the storm mean latitude and longitude point composited across all cases for each experiment group.

Comparing panels, the SM is different in both magnitude and spatial structure within the MCSarea. The southwestern quadrant of the PB‐1 experiment is predominantly dry, transitioning into wetter soils toward the north, east, and northeastern quadrants, with a total ∆SM ∼ 15% across the domain. The drier portions of the domain (and most places with SM < 25%) are also significantly different in magnitude with respect to the Control composite group, due to many places within the Control domain having SM ≥ 30%. This heterogeneous structure found in the PB‐1 experiments is like what was depicted in the GK2021 at antecedent hours to initiation. It should be noted that Figure 3 represents the structure of SM at antecedent times (1300–1600 UTC average); the SM structure at initiation time is nearly identical with Figure 3, indicating that the salient features of the SM patterns depicted are persistent up through the time of MCS initiations, and that further drying or moistening of the domain did not occur in between morning and initiation time (a disparity to results shown in GK2021).

### 5.2 SM Influence on MCS Initiations Through L‐A Interactions

The above section implies that with respect to near‐surface and other atmospheric variables in the PB‐1 experiment group, these values could be influenced by the differing SM configurations in Figure 3. It is natural to then ask how MCS initiations occurred with respect to these conditions within the MCSarea, and to further establish a mechanism of interaction related to the land surface state. One way to trace the effects of SM on subsequent MCS initiations in weakly forced synoptic environments is to examine the causal links between SM‐P and to break down this feedback loop into individual components. The process chain of local L‐A coupling (hereafter LoCo; Santanello et al., 2018) quantifies such causal links. LoCo is based on the idea that sensible and latent heat flux partitioning modulates the energy available for convective development by influencing near‐surface atmospheric temperature and moisture content, which ultimately determine the properties and the subsequent depth of the planetary boundary layer (PBL). Santanello et al. (2018) describes LoCo symbolically in Equation 1.

(1) $$∆\text{SM}\;\to ∆{\text{EF}}_{\text{sm}}\;\to ∆T_{2m},{∆Q}_{2m}\to ∆\text{PBLH}\to ∆\text{ENT}\Rightarrow ∆P,\text{Clouds}$$

Equation 1: LoCo Process Chain

where ∆SM is the change in soil moisture, EF is evaporative fraction, which is a function of latent (LE) and sensible (H) heat fluxes at the surface (Equation 2),

(2) $$\text{EF}=\frac{\text{LE}}{\text{LE}+H}$$

Equation 2: Evaporative Fraction

$∆{\text{EF}}_{\text{sm}}$ is the change of surface fluxes, $∆T_{2m}$ is the change in 2m‐temperature, ${∆Q}_{2m}$ is the change in 2m‐humidity, (PBLH; ∆PBLH) is the change in planetary boundary layer height, ∆ENT is the changes of entrainment at the top of the boundary layer, and P is precipitation. With the goal of identifying a mechanism of interaction, the LoCo framework is loosely used in the discussion below.

**Figure 4:**
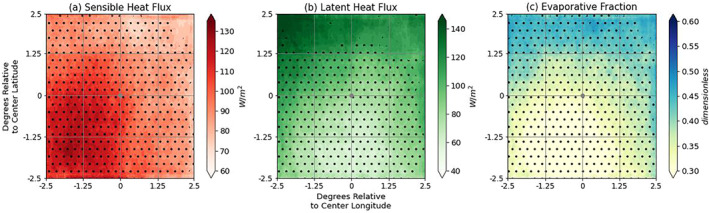
PB‐1 experiment (a) Sensible Heat Flux (H; W/m^2^), (b) Latent Heat Flux (LE; W/m^2^), and (c) Evaporative Fraction (EF) storm‐centered composites over each MCSarea, evaluated between 1300 and 1600 UTC. Stippling represents each variable's area that is significantly different from the Control sample at the 95% confidence level.

The first,a or “terrestrial,” leg of the LoCo process chain describes how changes of SM influence the sensible and latent heat flux partitioning at the surface, ultimately affecting the evaporation fraction values over the domain of interest. Figure 4 depicts three different storm‐centered composites for PB‐1: (a) sensible heat flux (H), (b) latent heat flux (LE), and (c) EF (Equation 2). Each field is evaluated during morning hours (1300–1600 UTC), which shows consistent results with the SM configuration as depicted in Figure 3. The dry portions of the PB‐1 domain in Figure 3 correspond with high amounts of H, but the main pattern depicted in the EF plot (Figure 4c) corresponds directly with the values of LE (reflecting Equation 2), which shows a similarly oriented S‐to‐N transition between drier (low LE) and wetter (high LE) soils.

Although SM and EF seem to be directly correlated with one another, it is worth noting there is an established nonlinear relationship that exists between these two variables; EF is a function of SM only across part of the range of SM (see Figure 5 in Seneviratne et al. (2010)). Defined by a piecewise function, there are two breakpoints: the wilting point, below which SM values are too dry (i.e., the “dry” regime), prohibiting evapotranspiration, and the critical SM value, above which LE dominates at the surface (i.e., the “wet” regime), and evapotranspiration becomes limited by net radiation and independent of SM. The “transitional” regime lies between these two breakpoints, wherein SM has the highest potential to influence near‐surface humidity and temperature, approximately linearly. The closer the wilting point and critical SM values are to one another, the larger the slope in the transitional regime (dEF/dSM) and the greater the sensitivity of the atmosphere to small variations in SM. Defining these points however is highly dependent on soil type, texture, vegetation, and climate, referring to “L‐A hot spots” where transitional zones are geographically found.

**Figure 5:**
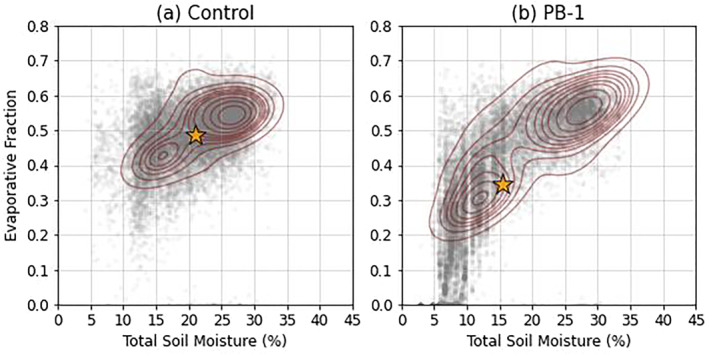
SM‐ EF plots for (a) Control and (b) experiment PB‐1, where all grid points within MCSarea are averaged to 0.1° × 0.1° boxes and then used in the sampling. Multivariate kernel density estimates are overlaid in red contours, and the yellow star represents the mean SM‐EF value in the sample.

To better determine what portion of each MCSarea lies within the transitional regime and to define the strength of the relationship between SM and EF for each experiment group, Figure 5 shows SM and EF plotted for every experiment, sampling all grid points in every MCS area during morning hours (1300–1600 UTC average). To declutter and simplify the diagram from 4 km × 4 km grid spacing, an average value of every 0.1° × 0.1° box inside of each MCSarea is plotted. A multivariate kernel density estimate (KDE) is also overlaid in red to help identify the clustering of points in the SM‐EF space, along with the mean SM‐EF point denoted by a star in each panel.

Although it is apparent that both the Control and PB‐1 experiments have different distributions of SM‐EF points, one distinct difference is the disparity of the two maxima in the KDE structure in Figure 5b, and how the mean SM‐EF value is reduced by ∆SM ∼ 5% and ∆EF ∼ 0.15 compared to Control. Compared to the referenced figure from Seneviratne et al. (2010), only PB‐1 samples SM values exhibit a linear relationship across the full range between the apparent wilting point and critical SM (∼5% and 15%, respectively), suggesting that there are more locations in the PB‐1 MCSareas that lie within the transitional regime. There are also two maxima in the Control group, and an apparent critical SM value exists (∼20%), implying that the Control MCSareas locations may be different from where the PB‐1 MCSareas are (this will be further discussed in Section 5.3).

**Figure 6:**
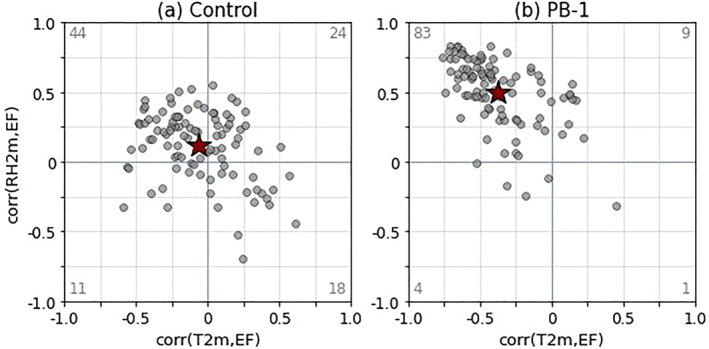
Correlation of 2m‐temperature (T2m) and EF plotted against the correlation of 2m‐relative humidity (RH2m) and EF, for (a) Control and (b) experiment PB‐1. Each axis is between −1 and 1 indicating the range of values for the Pearson correlation coefficient. The red star is the average correlation value in the correlation phase space. Gray number values represent the number of cases that fall into each corresponding quadrant.

If the samples in PB‐1 lie in areas of high L‐A coupling strength, this should be directly reflected in the correlation values of SM and near‐surface variables such as 2 m‐relative humidity (RH2m) and 2m‐temperature (T2m). Figure 6 shows the Pearson correlation coefficient between EF and T2m (corr(EF, T2m)) and the Pearson correlation coefficient between EF and RH2m (corr(EF, RH2m)), plotted against one another, with each coefficient representing the evaluation of correlation over the respective MCSarea, averaged over morning hours. For areas with a strong L‐A coupling strength, one would expect a high anticorrelation between corr(EF, T2m) (corresponding with low EF for higher T2m) and a high correlation between corr(EF, RH2m) (corresponding with high EF for higher RH2m). Out of the two plots shown, the PB‐1 sample best depicts this relationship, with the correlation values mainly falling within the upper left quadrant, and the average value is indicative that the corr(EF,RH2m) is strongest comparatively to corr(EF,T2m) (average value ∼0.5 vs. ∼0.4, respectively).

**Figure 7:**
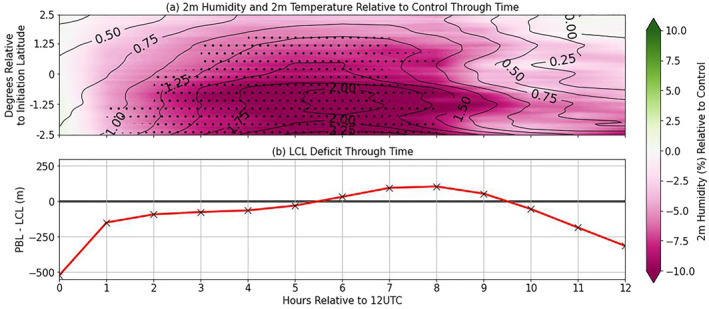
(a) SEA difference plots of 2m‐relative humidity (RH2m; shaded) and 2m‐temperature (T2m; black contour) with respect to Control, where the *y*‐axis is degrees relative to initiation latitude and (b) evolution of LCL deficit [PBLH‐LCL] in meters. For both (a) and (b), the *x*‐axis is hours relative to 1200 UTC. Stippling is representative for significant T2m values compared to the Control sample at the 95% confidence level.

Figure 6 is useful in determining the overall strength of the relationship between EF and near‐surface atmospheric variables; however, this is only a singular metric that does not convey if there is any coherent structure of these variables with relation to the SM structure depicted in Figure 3. The evolution of T2m and RH2m, based on SEA, displayed as the difference from Control (PB‐1—Control), is shown in Figure 7a. As a reminder, storm‐centered composites are zonally averaged and plotted through time to depict a latitudinal and temporal dependence of the variable of interest.

In the context of Figure 7a, where RH2m is shaded and T2m is contoured, the pink shades indicate less RH2m in comparison to Control, and vice versa for the green shades. Progressing through time (relative to 1200 UTC; initialization time), the subsequent drying out and warming of the MCSarea is shown, with the warmest and driest areas corresponding with the southern quadrants of the domain (matching with the drier SM values in the SW quadrant in Figure 3). The T2m and RH2m contours eventually form a north‐south structure at 1800 UTC (+6 hr) that reaches ≥2°C T2m and ≥10% RH2m departure from Control.

Figure 7b has the same *x*‐axis as Figure 7a (progression through time relative to 1200 UTC) but depicts the value of the lifted condensation level (LCL) deficit, defined as the difference between PBL height (PBLH) and LCL height [PBLH–LCL], which is a necessary condition for deep convection. In Figure 7b, the triggering of deep convection occurs on average at 1800 UTC (6 hr after 1200 UTC), which coincides with the maximization of the T2m and RH2m surface gradient shown in Figure 7a. The conditions that eventually lead to the PBL crossing the LCL (related to the land surface fluxes) are ultimately defined by different mechanisms dependent on the abundance of either H or LE at the surface. In this particular case, the drying and heating of the surface over time is associated with an abundance of surface H, mainly in the southern quadrants of the MCSarea (viz. Figure 4). This dominant process can be described by more heat being transported into the atmosphere and an increase in near‐surface temperature, leading to rising motion (from decreased density and expansion of air immediately next to the surface), driving the PBL upward and meeting the defined LCL height to trigger deep convection.

**Figure 8:**
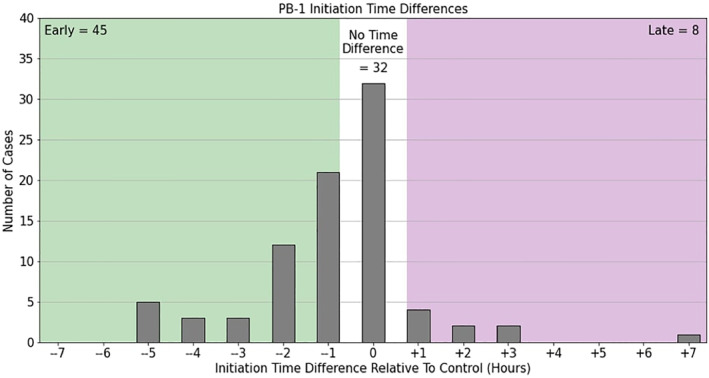
Histogram of PB‐1 MCS initiation time distribution compared to Control. Each bin unit is in hours relative to Control.

Characterizing the structure of mean near‐surface variables and their average evolution is useful, but what evidence is there to suggest that this near‐surface configuration is influencing the timing of deep convection, and more importantly, does this timing differ from Control simulations? Figure 8 provides a histogram of case counts for the initiation time difference from Control in hours, where all PB‐1 events binned in the green area occur before the corresponding Control initiation (i.e., earlier in time), and all PB‐1 events binned in the purple area occur after the corresponding Control initiation. Although about one‐third of PB‐1 MCS events were triggered at the same time as the Control initiation (*n* = 32), more than half of cases (*n* = 45) that resulted in an initiation were triggered earlier, on average occurring between 1 and 2 hr before the Control initiation. A smaller subset of cases (*n* = 8) were triggered after the Control initiation. This result indicates that the implementation of the PB‐1 experiment in the land initial conditions produces sufficient changes such that the imposed perturbation can affect the timing of MCS. Quantifying the median SM heterogeneity between these three case groups (depicted in Figure S2 of the Supporting Information S1) shows that within these samples, larger SM gradients (∼5%) were prevalent in the early onset initiations of MCSs in this sample compared to Control.

**Figure 9:**
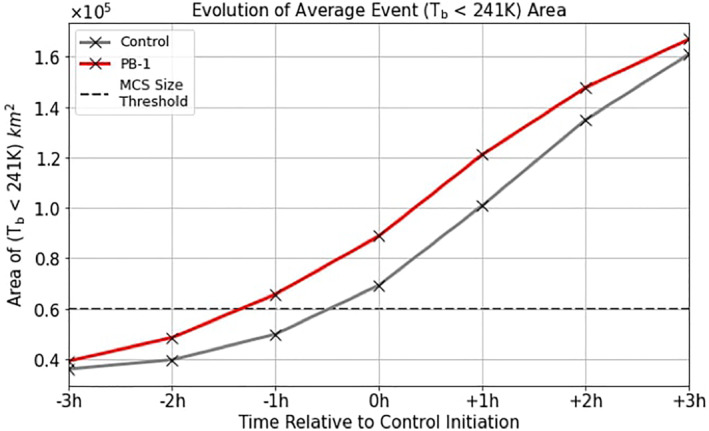
Average CCS area for MCS events in each experiment plotted with respect to the Control initiation time. The MCS size threshold (0.6 × 10^5^ km^2^) is represented by the dashed line.

Another aspect related to the timing of each event is when the size criterion for MCS is met. In observations (MCS2019), this is a CCS with a size of at least 6 × 10^4^ km^2^. The size of the PB‐1 MCS events was tracked over time and compared to Control. By depicting the count of pixels that meet the CCS threshold (*T*_*b*_ < 241K) (within a 7.5° × 7.5° domain surrounding the recorded initiation), Figure 9 shows the progression of event size on average, with respect to the Control initiation time rather than 1200 UTC (to better frame the relevant timeframe of when the CCS size threshold is met). Consistent with Figure 8, the PB‐1 experiment reaches this MCS size threshold between one and 2 hr before the initiation time; however, this figure also shows that the CCS grows larger (+0.2 × 10^5^ km^2^) than the Control CCS for the three‐hour period beyond the Control initiation, further indicating the imposed perturbation affects the timing of MCS events.

Using the LoCo framework to summarize the results above, the average underlying SM conditions in the PB‐1 experiment group introduced a chain reaction from the SM heterogeneity structure (viz. Figure 3) imposing changes to near‐surface thermodynamic variables, which affected the rate of growth of the PBL, and subsequently the timing of deep convection and meeting the MCS size threshold. Referring to Figures 8 and 9 in particular, it is natural to wonder how the dynamical response of the atmosphere is affected by the average SM configuration in the PB‐1 experiment group, considering the events are occurring earlier in time, and the average CCS is larger compared to Control.

**Figure 10:**
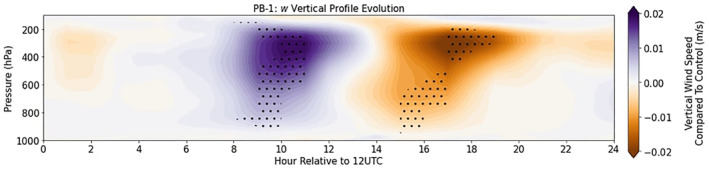
SEA difference plot of vertical velocity (*w*) profile with respect to Control. The *y*‐axis is height expressed as pressure (hPa) and the *x*‐axis is hours relative to 1200 UTC. Stippling shows significant *w* values compared to the Control sample at the 95% confidence level.

The time evolution of the vertical profile of vertical velocity (*w*) is a measure of the amplification or suppression of upward motion compared to the Control events on average. Using SEA and differencing [PB‐1–Control], Figure 10 depicts the evolution of spatially averaged *w* (within a 5° × 5° domain surrounding the recorded initiation) relative to initialization (1200 UTC), where purple shades represent larger *w* compared to Control, and orange shades represent smaller *w* compared to Control. About 8–9 hr after 1200 UTC (2000–2100 UTC/1500–1600 LST) significantly larger *w* is seen throughout the atmospheric column compared to Control, with the most significant differences occurring in the upper levels of the troposphere (above ∼500 hPa). Progressing through time, this increase of *w* transitions into significantly smaller *w* throughout the atmospheric column, again with the most significant values above 500 hPa. This dipole‐like structure of *w* compared to Control emphasizes a shift in time of the actual event on average, rather than an increase in overall vertical velocity for the entirety of the storm duration which would indicate that the storms on average are longer lasting.

**Figure 11:**
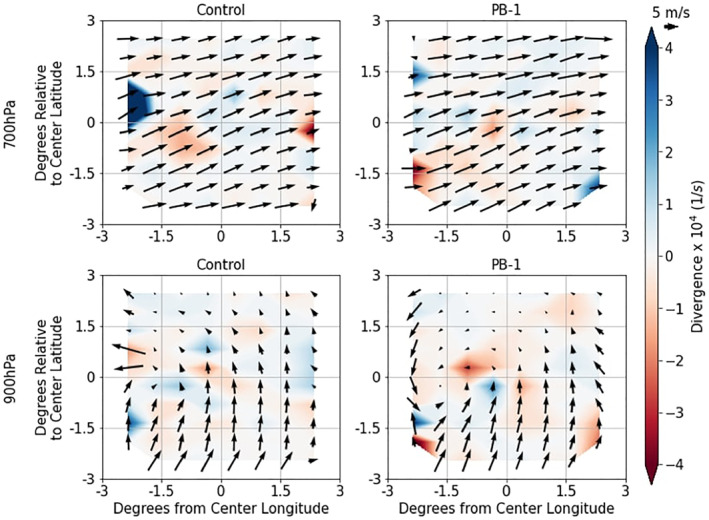
700 hPa (top row) and 900 hPa (bottom row) *u*‐*v* winds (m/s) at (Initiation—1 hr) time as a function of degrees longitude and latitude from the center initiation point for Control experiments (left column) and PB‐1 experiments (right column). Shaded contours represent storm‐centered composite divergence (×10^4^ 1/s).

One important portion of the dynamical response related to the imposed SM perturbation is how the near‐surface wind field is oriented to the SM heterogeneity (viz. Figure 3b). Looking at both near surface (900 hPa) and above the boundary layer (700 hPa) for both experiments, a storm‐centered composite of divergence (×10^4^ 1/s) and *u*‐*v* wind field (m/s) is created for both Control and PB‐1, shown in Figure 11. The timing for this plot was chosen to be 1 hr before recorded initiation to isolate the antecedent conditions before any mesoscale wind fields associated with the MCS perturb the local wind field.

At the 700 hPa level, there are few differences between the two experiments, with both wind fields flowing primarily west‐southwesterly, and slightly stronger winds in the PB‐1 experiment. At the near‐surface (900 hPa), however, larger differences exist between the two groups that show the overall wind field in PB‐1 is aligned with the organized SM heterogeneity in Figure 3b. The wind fields from both experiment groups show south‐southwesterly flow in the southern portions of the domain; however, within the context of PB‐1, this flow is oriented parallel to the SM heterogeneity gradient direction (Figure 3b), consisting of drier SM in the southwest and wetter soil in the northeastern portions of the MCSarea. Along with the overall increased convergence (shaded red) in the PB‐1 storm‐centered composite at 900 hPa, the *u*‐*v* wind field also exhibits organized convergence in the form of cyclonic rotation at variable wind speeds (ranging between less than 5–10 m/s), with the center of this rotation located on the drier side near the edge of the SM heterogeneity pattern depicted in the MCSarea, and veering with height which is associated with dynamical lifting and warm air advection. It should be noted that the wind field at antecedent morning time hours was also assessed and shows similar features, with the exception of less developed cyclonic rotation at lower levels, and less veering with height (southwesterly flow at 900 hPa level rather than south‐southwesterly). These results are like the findings of GK2021 that showed low‐level wind fields flowing parallel to the SM heterogeneity gradient direction in observational analyses.

### 5.3 How Does SM Influence the Location of MCS?

Comparing the low‐level wind field of the PB‐1 experiment (Figure 11) to the mean storm‐centered SM composite at initiation time (Figure 3b), shows that on average, the convergence and upward motion coinciding with MCS initiation events occur over the drier portions of a dry‐to‐wet SM gradient. It is still unknown, however, if the individual location departures from the center of the EXParea (i.e., the observational MCS location) can be described with respect to the hypothetical mechanism of interaction implied in T2011 and GK2021, and if the implemented dry SM perturbation in the model (which introduced drier‐to‐wetter SM gradients along the edges of the perturbed area) has any effect on the location of the cases.

**Figure 12:**
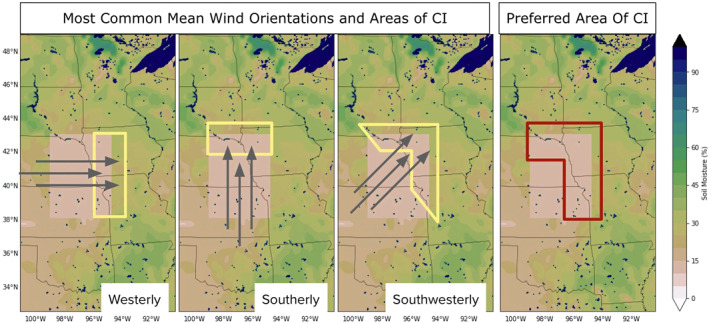
Schematic depicting the most common mean wind orientations in the PB‐1 experiment and the preferred regions for convective initiation (CI) outlined in yellow for three categories of wind orientations, followed by a panel (rightmost) that depicts the cumulative preferred region of CI outlined in red. The location for the PB‐1 experiment in this example is for an arbitrary MCS case; background geography is provided for scale.

This relationship, with respect to the alignment of the mean low‐level wind to the SM gradient direction, can be described in such a way that considers common mean wind orientations in the Great Plains region, creating a hypothetical preferred area of convective initiation (CI) based on those directions. While low‐level winds are variable, the occurrence of the GPLLJ and dominant westerly flow over the Rocky Mountains suggests that the most common mean wind orientations would be a combination of westerly, southerly, and southwesterly flows. In Figure 12, the area of CI is outlined in yellow, where each scenario describes the hypothetical mechanism of SM heterogeneity aligned with low‐level winds producing increased areas of convergence and lift to generate MCS initiations. Combining these three scenarios produces one preferred area of CI for the PB‐1 cases (outlined in red in the rightmost panel), which is centered in the northern, eastern, and northeastern portions of the defined EXParea, and encompasses more areas of the PB‐1 perturbation to account for the fact that in past studies, initiations were preferred over the drier portions of SM heterogeneity.

**Figure 13:**
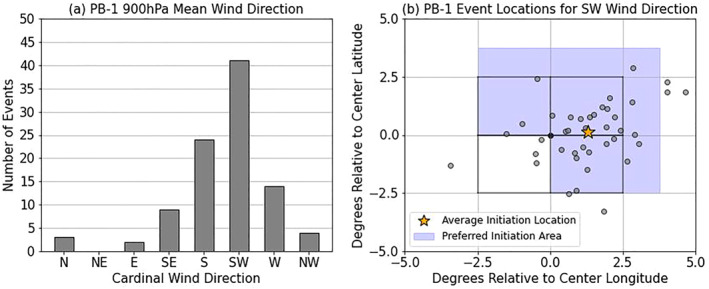
(a) Histogram of early morning (1300–1600 UTC average) mean 900 hPa wind direction, evaluated over each MCS area for PB‐1, and (b) CI points from cases with a southwesterly (SW) mean 900 hPa wind with respect to the EXParea center (defined as the 5° × 5° area of the experiment placement), where the preferred area of initiation outlined in Figure 12 is shaded blue. The average CI point is denoted by the star.

To verify this hypothesis, the low‐level (900 hPa) mean wind direction in the early morning (1300–1600 UTC) was assessed (Figure 13a) over each implemented EXParea in the model, organized into a histogram showing the frequency of each cardinal and intercardinal wind direction during that period. The overwhelming majority of cases (∼85%) fall into the three most common mean wind orientations outlined in Figure 12, with over half of all cases having a southwesterly mean wind in the morning time hours. The southwesterly mean wind cases (*n* = 44) were plotted with respect to where the recorded MCS initiation occurred with respect to the center of the EXParea, depicted in Figure 13b. Over 75% of the cases in this category occur within the hypothesized preferred area of CI (shaded in blue), with most of those cases occurring on the dry side of the SM gradient. In comparison, the recorded MCS cases in the Control case had a higher standard deviation with respect to the average distance from the EXParea center. These results suggest that the impact of the SM heterogeneity (introduced through the implementation of PB‐1 experiments) is nonnegligible and that with respect to the prevailing wind direction, drier soils upstream and wetter soils downstream can influence the location of MCS events in these simulations.

## 6 Conclusions and Discussion

The aim of this work has been to explore if and how summertime MCSs in the US Great Plains, occurring in weakly forced synoptic environments, are influenced by SM heterogeneity. Using WRF to perform two‐way nested convective‐permitting simulations, two experiment groups were carried out (Control/unaltered SM and PB‐1). Inspired by T2011, BKC2018, and GK2021, the experimental setup of the PB‐1 simulations consisted of a 5° × 5° patch of uniform near‐wilting point SM centered at the location of a weakly forced observed MCS event specifically chosen from an MCS event catalog (MCS2019; Feng et al., 2019). A total of 97 separate cases were simulated. Each simulation spans a 24‐hr period beginning at 1200 UTC on the day of the observational MCS event, and the imposed SM perturbation in the PB‐1 experiment group is allowed to evolve with the duration of each run (i.e., only imposed in the initial conditions). The PB‐1 experimental setup was created to observe the effect of a SM perturbation on the location and timing of MCS events comparatively to Control simulations, and to establish whether or not a mechanism could be identified that supports the hypothesis that the alignment of the background wind to a SM heterogeneity pattern (such that dry patches are upstream and wet patches are downstream) induces convergence and upward motion through meso‐β circulations to support the initiation of weakly forced MCS.

A storm‐centered composite analysis of SM at the 5° × 5° scale showed that in the PB‐1 experiments, SM heterogeneity [O(100) km] structured as significantly drier soils to the southwest (SW) of the mean initiation, transitioning to wetter soils northeast (NE) of the mean initiation, was underlying the recorded WRF MCS events. The SM influenced surface fluxes, such that a gradient of *H is* formed (with higher *H* in the southwestern portions and lower *H* in the northeastern portions of the domain). A large sample of the SM points in the PB‐1 storm‐centered composites had values that lay within the transitional regime (i.e., when SM and EF changes have a positive linear relationship), whereas the Control group did not support the existence of a relationship of near‐surface variables to the SM pattern in the PB‐1 storm‐centered composite.

It was found that a gradient of 2m‐temperature and 2m‐humidity, aligned south‐to‐north coinciding with the zonal average of the SM configuration, strengthened in magnitude over the duration of the run, which coincided with the growth of the planetary boundary layer to eventually trigger MCSs at the time of maximum 2m‐temperature and 2m‐humidity gradient. Consequently, the tracked events occurred earlier in time (∼1–2 hr on average) compared to Control simulations. This was further supported by the dynamic variable changes, where a dipole‐like shift of the vertical wind profile in time compared to Control suggested earlier onset of initiations. It was also found that the near‐surface wind field was aligned in a way consistent with previous findings, where the mean *u*‐*v* wind field is oriented with drier SM upstream and wetter SM downstream, accompanied by veering of wind with height. Cyclonic rotation of the wind field at the 900 hPa level resulted in more widespread convergence in the storm‐centered composite domain compared to Control.

Assessing the overall impact of the spatiotemporal changes of MCS events with respect to the imposed SM heterogeneity was hypothesized through the lens of past work (T2011, GK2021). The implementation of the dry SM perturbation in the model domain, which introduced drier‐to‐wetter SM gradients along the edges of the perturbed area, was predicted in the context of the overall findings to influence the location of MCS initiations relative to the center of the perturbed area (i.e., the observational MCS). Assessing the low‐level wind field in morning showed that MCS initiations were subsequently preferred on the dry side of the transition zones created by the perturbation, with the most common orientation of simulated MCS events on average initiating in the northern and eastern quadrants of the PB‐1/SM perturbation, embedded within southwesterly flow. This narrative supports the resulting‐organized SM heterogeneity depicted in the mean storm‐centered composites at initiation time (i.e., centered in space and time at each simulated MCS initiation point) from the PB‐1 experiment group. These results suggests that with respect to the orientation of the low‐level wind, drier soils upstream and wetter soils downstream can influence the location of MCS events, and that meso‐β circulations related to near‐surface variable fluctuations can be used to describe the main mechanism of interaction of weakly forced synoptic events in these simulations.

With these results, future work avenues could delve deeper into sensitivity studies that asses the sign and strength of the SM‐P coupling found here, which can directly address the known model‐dependency results that could stem from parameterization choice (e.g., Feng et al., 2018), scale choice (e.g., Albertson et al., 2001), and including or excluding convective parameterization (e.g., Done et al., 2006; Prein et al., 2020). The experimentation of tracking algorithms to define MCS case samples, or spectral analyses to better define subjective values where the length scale of organized SM heterogeneity is most influential, would also help support the findings in this study and in this hot‐spot region.

## Supporting information

Supporting Information S1

## Data Availability

ERA‐5 data analyses are generated using Copernicus Climate Change Service information (2004–2017), accessible at https://cds.climate.copernicus.eu; neither the European Commission nor ECMWF is responsible for any use that may be made of the Copernicus information or data it contains. MCS events in this study are openly available at https://doi.org/10.5439/1571643 as cited in Feng et al. (2019). Software necessary to reproduce the results of this paper include WRF‐ARW version 4.3.1, which is available for download at https://github.com/wrf‐model/WRF/releases/tag/v4.3.1. Model data used to produce the figures and tables, along with analysis codes and example model namelists are available at https://doi.org/10.13021/orc2020/Y2MYGB.
