# Supplementary material for: Identifying the Mechanism of Interaction Between Soil Moisture State and Summertime MCS Initiations in Weakly Forced Synoptic Environments Using Convective‐Permitting Simulations
Source: J Geophys Res Atmos. 2024 Dec 1;129(23):e2024JD040855. doi: 10.1029/2024JD040855 (PMC11608822; doi:10.1029/2024JD040855)
Supplement: Supplementary file 1 — Supporting Information S1 [file JGRD-129-0-s001.pdf]

Journal of Geophysical Research: Atmospheres

Supporting Information for

Identifying the Mechanism of Interaction Between Soil Moisture State and Summertime MCS Initiations In Weakly-Forced Synoptic Environments Using Convective-Permitting Simulations

Rachel Gaal[1], James L. Kinter III[2], Paul A. Dirmeyer[2], and Bohar Singh[3]

[1] Department of Atmospheric Science, Rosenstiel School of Marine and Atmospheric Science, University of Miami, Miami, FL

[2] Center for Ocean–Land–Atmosphere Studies, George Mason University, Fairfax, Virginia

[3] International Research Institute for Climate and Society, Columbia University, Palisades, New York

Contents of this file

Figures S1 to S2

Introduction

Supporting information in this document includes supplementary figures that help describe the methodology and results of this manuscript.

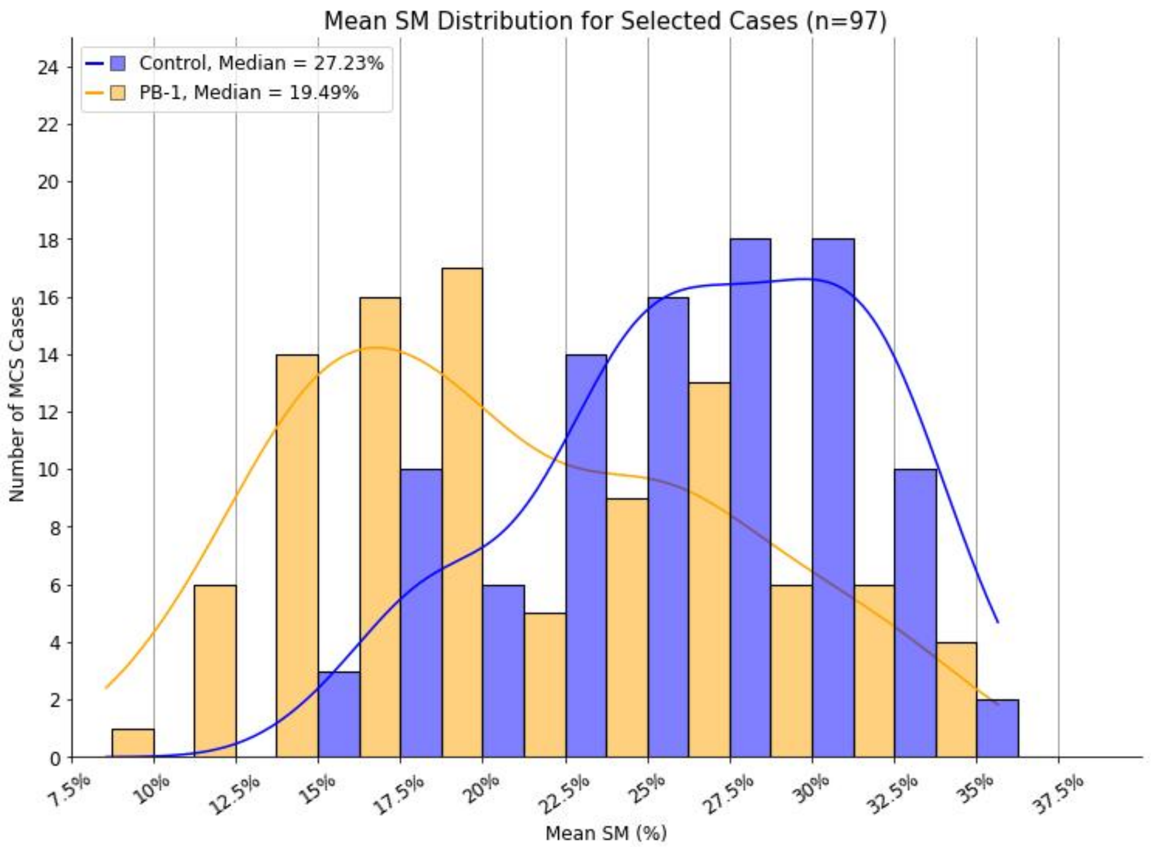

Figure S1. Control and PB-1 mean SM distributions for selected cases in experimental setup. Histogram bars represent the number of MCS cases within the mean SM value (%) bin (2.5% increments), and the solid curve represents a density estimate for the sample group. Median SM values are given for each sample group within the legend.

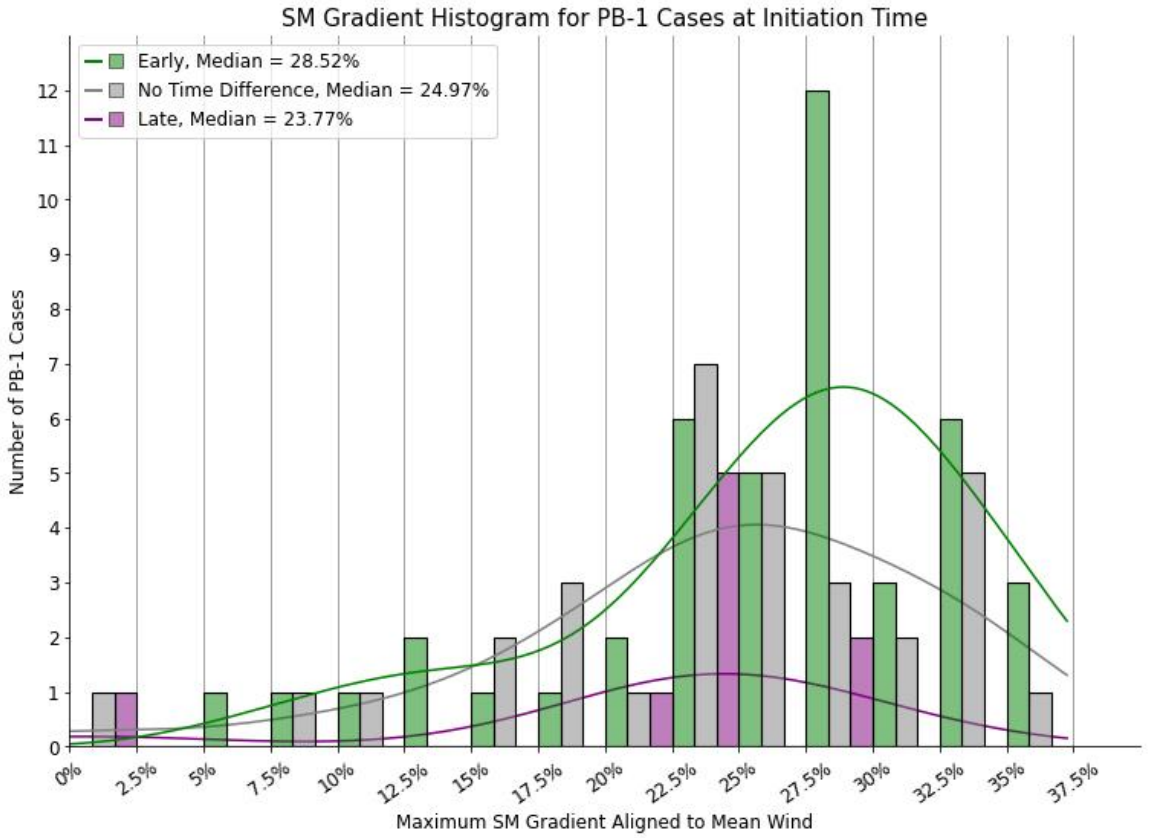

Figure S2. Histogram of PB-1 maximum SM gradients aligned to the mean wind, color categorized by their timing relative to the Control initiation. For each category, the relevant initiation cases were identified by location and prevailing morning-time winds. The SM gradient is calculated as the difference between the maximum and minimum SM values (in the 5° X 5° analysis area) that was aligned with the identified wind direction, such that the minimum SM value was flagged upstream of the prevailing wind, and conversely, the maximum SM value was flagged downstream of the prevailing wind. For example, an MCS that was identified to occur earlier in time than Control with a mean prevailing southwesterly wind (i.e. flowing SW-to-NE), the minimum (driest) SM value is evaluated in the SW quadrant of the area surrounding the identified model initiation point, and the maximum (wettest) SM value is evaluated in the NE quadrant. To remove the possibility of the minimum and maximum grid points being identified at a scale that is too close in distance to one another (to try and examine gradients with at least [O(100km)]), we only evaluated the combination of maximum and minimum SM values that had at least 100 km of Euclidean distance between them. Histogram bars represent the number of MCS cases within the mean SM value (%) bin (2.5% increments), and the solid curve represents a density estimate for the sample group. Median SM gradient values are given for each sample group within the legend.
